# Epidemiology of Shuni Virus in Horses in South Africa

**DOI:** 10.3390/v13050937

**Published:** 2021-05-19

**Authors:** Thopisang P. Motlou, June Williams, Marietjie Venter

**Affiliations:** 1 Zoonotic Arbo and Respiratory Virus Program, Centre for Viral Zoonoses, Department Medical Virology, Faculty of Health Sciences, University of Pretoria, Pretoria 0031, South Africa; thopisangmotlou@gmail.com; 2 Department of Paraclinical Sciences, Section Pathology, Faculty of Veterinary Science, University of Pretoria, Pretoria 0110, South Africa; june.williams@up.ac.za

**Keywords:** epidemiology, horse, neurological disease, orthobunyavirus, RT-PCR, Shuni virus, South Africa

## Abstract

The *Orthobunyavirus* genus, family *Peribunyaviridae*, contains several important emerging and re-emerging arboviruses of veterinary and medical importance. These viruses may cause mild febrile illness, to severe encephalitis, fetal deformity, abortion, hemorrhagic fever and death in humans and/or animals. Shuni virus (SHUV) is a zoonotic arbovirus thought to be transmitted by hematophagous arthropods. It was previously reported in a child in Nigeria in 1966 and horses in Southern Africa in the 1970s and again in 2009, and in humans with neurological signs in 2017. Here we investigated the epidemiology and phylogenetic relationship of SHUV strains detected in horses presenting with febrile and neurological signs in South Africa. In total, 24/1820 (1.3%) horses submitted to the zoonotic arbovirus surveillance program tested positive by real-time reverse transcription (RTPCR) between 2009 and 2019. Cases were detected in all provinces with most occurring in Gauteng (9/24, 37.5%). Neurological signs occurred in 21/24 (87.5%) with a fatality rate of 45.8%. Partial sequencing of the nucleocapsid gene clustered the identified strains with SHUV strains previously identified in South Africa (SA). Full genome sequencing of a neurological case detected in 2016 showed 97.8% similarity to the SHUV SA strain (SAE18/09) and 97.5% with the Nigerian strain and 97.1% to the 2014 Israeli strain. Our findings suggest that SHUV is circulating annually in SA and despite it being relatively rare, it causes severe neurological disease and death in horses.

## 1. Introduction

The *Orthobunyavirus* genus, family *Peribunyaviridae* belongs to the order *Bunyavirales* which consists mostly of vector-borne viral diseases transmitted by arthropods (e.g., mosquitoes, ticks, flies and midges) [1]. The viruses in this order produce illnesses ranging from acute febrile to hemorrhagic fevers, neuroinvasive diseases, abortions and birth defects [2,3].

The *Peribunyaviridae* family are negative sense single stranded RNA (ss-RNA) viruses with a segmented genome [4,5]. The small (S) and large (L) segments encode the nucleocapsid and polymerase proteins respectively and are highly conserved, while the medium (M) segment encodes a polyprotein consisting of two glyoproteins and a nonstructural protein that is highly variable [6,7]. Genetic reassortment occurs naturally within these viruses due to the segmented genome leading to the emergence of new viruses in which the pathogenicity may be increased [8]. Viruses in the *Orthobunyavirus* genus are widely distributed in different continents [7,9] and include viruses of significant importance such as Akabane [6], Schmallenberg [10,11] and Oropouche viruses which have been reported to cause significant clinical diseases variability in humans and animals [12].

In this study we focused on the orthobunyavirus, Shuni virus (SHUV), a neglected re-emerging zoonotic virus thought to be transmitted by hematophagous arthropods. Shuni virus was first isolated from a cow in Sokoto, Nigeria in 1966 [13]. The only reported human case was from a febrile child in Nigeria in 1966 [14]. The virus was later recovered twice in South Africa (SA), from mosquitoes (*Culex theileri*) and healthy cattle in 1972 [15]. It has also been isolated from the brains of two horses, one from South Africa (SA) and the other from Zimbabwe, in the 1970s [15,16] but not further investigated again until 2009 when surveillance for neurological arboviruses in horses was implemented by the Zoonotic arbo- and respiratory virus (ZARV) group, University of Pretoria (UP), SA. The investigation led to the detection of SHUV in 6% of the horse cases which displayed severe neurological and febrile signs with an overall fatality rate of 42.9% [17]. Antibodies against SHUV were subsequently identified in 3.9% of veterinarians involved in equine, wildlife and livestock services in South Africa suggesting that human exposure may occur [18]. We recently reported SHUV in humans with neurological signs in hospitals in the Gauteng Province. SHUV was detected in 5% of CSF samples from cases of unsolved neurological signs in patients [19]. This increases the importance of defining SHUV’s epidemiology as a potential zoonotic virus in Africa.

SHUV was also identified in malformed ruminants during an outbreak in Israel between 2014 and 2015 and neurological infections in cattle in 2019 suggesting that SHUV may have the capacity to emerge in new regions [20,21]. Detection of SHUV in *Culicoides* midges and *Culex theileri* mosquitoes has been reported in an historical vector survey in Africa [22] and recent vector competence studies in Europe suggested that *Culicoides* biting midges are competent vectors when compared to *Culex pipiens pipiens*, and *Aedes aegypti* mosquitoes [23].

Despite SHUV being detected on several occasions in Africa in the last 50 years, little is known about its epidemiology, its reservoir host/s and importance in human or animal disease. Therefore, the purpose of this study was to define the epidemiology of SHUV in SA by investigating its association with febrile and neurological disease in horses over a period of 10 years. Phylogenetic analysis of identified strains and full genome sequencing of a recent isolate allowed us to describe the molecular epidemiology of circulating SHUV strains in horses in South Africa. The analysis of the incidence of SHUV-associated disease, clinical presentation, seasonality and geographic distribution of SHUV gave insight into the epidemiology.

## 2. Materials and Methods

### 2.1. Sampling

This study was part of a surveillance program for zoonotic arboviruses that may cause febrile and neurological diseases in horses and other animals. Horse specimens were screened for the presence of SHUV between 2013 and 2019 and compared to previously detected SHUV positive cases between 2009 and 2012 as described in van Eeden et al. 2013 [17]. The specimens were submitted by veterinarians from across SA to the ZARV program as part of a passive surveillance program. All cases were accompanied by a submission form with detailed demographic and clinical information. All specimens received were processed in a biosafety level 3 (BSL3) laboratory at the Centre for Viral Zoonoses (CVZ), Department of Medical Virology, University of Pretoria, SA. The samples are stored at −80 °C in the CVZ biobank.

Ethical clearance was granted by the University of Pretoria Faculty of Health ethics committee. Animal ethical approval was also granted for the screening of horse samples in this study: Project number: H001-17. Section 20 approval was obtained from the Department of Agriculture, Forestry and Fisheries (DAFF) as part of the Surveillance for neurological arbovirus study (12/11/1/1) 2016 and 2019. The samples are stored at −80 °C at the Centre for Viral Zoonoses, University of Pretoria sample repository as part of the BSL3 BioBank.

### 2.2. Sample Extraction

Nucleic acid extractions from serum, plasma, and whole blood were conducted using the QIAamp Viral RNA Kit (Qiagen, Hilden, Germany). Samples were placed in lysis buffer and extracted in the BSL3 laboratory at the CVZ, UP.

### 2.3. The Orthobunyavirus TaqMan Real-Time RT-PCR Assay

The orthobunyavirus TaqMan real-time (RT-PCR) assay was conducted according to the in-house designed protocol [24]. The PCR was performed using the Agpath-ID One Step RT-PCR (Thermo Fisher Scientific, Waltham, MA, USA) according to the manufacturer’s instructions. All the primer and probes used in this assay are detected on ORF3 of the SHUV orthobunyavirus S segment. Briefly 10 µL of RNA was added to 0.45 µL of 20 µM primer (OrthoB1F (TAGAGTCTTCTTCCTCAAYCAGAAGAAGGCC)), OrthoB1R (GTYAMGGCAMTGTCTGGCACAGGATTTG)), 0.25 µL of 12 µM probe (OrthoB1P (TGGTTAATAACCATTTTCC)), 12.5 µL of 2X Ag-Path reaction mixture and 1 µL of 25X Ag-Path enzyme mixture to a final volume of 25 µL. The PCR reaction cycle consisted of 50 °C for 30 min and 94 °C for 2 min, followed by 40 cycles of 94 °C for 15 s and 58 °C for 1 min. Analysis was based on amplification plots and Ct values.

### 2.4. The SHUV S-Segment Conventional Nested PCR

The SHUV S-segment conventional nested PCR included a cDNA step followed by two rounds of PCR. First strand cDNA synthesis was conducted using the RevertAid RT kit (Thermo Fisher Scientific, Waltham, MA, USA) according to the manufacturer’s instructions. The first and second rounds were conducted using the Invitrogen Platinum Taq DNA polymerase kit (Invitrogen, Thermo Fisher Scientific, Waltham, MA, USA), according to the manufacturer’s instructions using primers previously described [25]. The nested real-time RT-PCR previously described [25] was used for SHUV screening from 2009 until 2017 when we switched to the Orthobunya TaqMan RT-PCR and only used the conventional version for obtaining larger regions for sequencing. Briefly 5 µL cDNA was added to 0.5 µL of each 20 pmol first-round primers (Forward primer: ShuS + (CGATACCGTTAGAGTCTTCTTCC) and reverse primer: ShuS − (CGAATTGGGCAA GGAAAGT)), 2.5 µL 10X HiFi buffer (Invitrogen, Thermo Fisher Scientific, Waltham, MA, USA), 1 µL MgCl_2_ (50 Mm), 0.5 µL of dNTP mix (10 mM), 0.1 µL 1X Platinum Taq DNA polymerase (Invitrogen, Thermo Fisher Scientific, Waltham, MA, USA), and nuclease free water to a final volume of 25 µL. The PCR cycle reaction began at an incubation of 94 °C for 2 min, followed by 35 cycles of 94 °C for 30 s, 55 °C for 1 min (50 °C for 1 min for second round) and 72 °C for 1 min and a final extension of 72 °C for 7 min.

### 2.5. Sequencing

Sequencing was performed using the BigDye Terminator v3.1 kit (Applied Biosystems, Foster City, CA, USA) according to the manufacturer’s instructions. Raw sequences obtained in an ABI format from the sequencing facility were resolved manually and assembled into a contig using the BioEdit DNA sequence alignment editor v7.0.5.3 software [26] and aligned using the ClustalW multiple alignment tool [27].

### 2.6. Virus Isolation

The virus isolation method was modified and adapted from Leland and Ginocchio, 2007 [28]. Briefly, processed whole blood, plasma, serum, CSF or suspensions of ground tissue were inoculated in African green monkey kidney (Vero) and baby hamster kidney fibroblasts (BHK) (ATCC, Manassas Historic District, VA, USA) cell lines. The cells were grown in Eagle’s medium supplemented with 10% (for Vero cell lines)/20% (for BHK cell lines) fetal calf serum (FCS) (Gibco, Thermo Fisher Scientific, Waltham, MA, USA) using 25 cm^3^ plastic tissue culture flasks (Greiner Bio-one, Kremsmünster, Austria). The inoculated flasks were incubated at 37 °C and 5% CO_2_ for a period of up to 7 days. Specimens showing >75% CPE within seven days following inoculation were considered positive and thus the cells were harvested and tested on PCR for the presence of SHUV. The supernatant fluid from specimen that did not show CPE after 7 days were subcultured twice more.

### 2.7. Full Genome Amplification Using Illumina MiSeq

Only SHUV positive samples that were successfully isolated were subjected to next generation sequencing (NGS). Three culture flasks were grown to 70% cytopathic effect (CPE). The supernatant was filtered through a Ministat single use filter unit 16555-K 0.45 filter (Sartorius Stedim Biotech, Göttingen, Germany) to remove possible bacterial contamination. The filtered product was concentrated with a Millipore Amicon Ultra-15 Centrifugal Filter Units 10K (Merck, Darmstadt, Germany) which excludes particles smaller than 10,000 Da. The concentrate was subjected to RNA extraction using Direct-zol RNA Miniprep with a DNAse step (Zymo Research, Irvine, CA, USA) and purified with the RNA Clean and Concentrator-5 kit (Zymo Research, Irvine, CA, USA).

This was followed by cDNA preparation using the sequence-independent, single-primer amplification and rapid amplification cDNA ends (SISPA-RACE) PCR as described in [29]. The prepared cDNA was sent to the National Institute for Communicable Diseases (NICD) for NGS using the Illumina MiSeq platform. Full de novo genome reconstruction was achieved by sequence assembly using the CLC sequence viewer (version 8.0). The full genome was reconstructed using SHUV reference sequences and Simbu serogroup reference sequences.

### 2.8. Phylogenetic Analysis

The edited consensus sequences were aligned using multiple alignment using fast Fourier transform (MAFFT) version 7.3.1.3 [30], a multiple sequence alignment program, with reference strains of viruses belonging to the Simbu serogroup of the *Peribunyaviridae* family. Initial tree(s) for the heuristic search were obtained automatically by applying Neighbor-Join and BioNJ algorithms to a matrix of pairwise distances estimated using the maximum composite likelihood (MCL) approach, and then selecting the topology with superior log likelihood value. A discrete Gamma distribution was used to model evolutionary rate differences among sites (5 categories (+*G*, parameter = 1.1155)). The rate variation model allowed for some sites to be evolutionarily invariable ([+*I*], 20.91% sites). The tree was drawn to scale, with branch lengths measured in the number of substitutions per site. The analysis in this study involved 39 nucleotide sequences. All positions containing gaps and missing data were eliminated (complete deletion option). There was a total of 327 positions in the final dataset. The sequences were subjected to phylogenetic analysis using the Molecular Evolutionary Genetics Analysis (MEGA) X [31] software by generating a maximum likelihood tree at a bootstrap value of 1000 replicates. Nucleotide genetic similarity analysis was performed to determine the similarity of the newly sequenced products with sequences from the Simbu serogroup (based on phylogenetic analysis). Nucleotide similarity matrices were generated from the alignment in MEGA and exported into Excel (Microsoft, Redmond, WA, USA) for further analysis.

## 3. Results

RT-PCR screening of samples from 1820 horses with febrile and/or neurological signs identified 24 (1.3%, Range 0.7–3.4% per year) SHUV positive cases between 2009–2019 (Supplementary Table S1). A SHUV-specific HyProbe nested real-time RT-PCR [25] was initially used for screening which detected 19/24 (79.2%) of the SHUV positive cases between 2009 and 2016. The screening assay was subsequently changed to an *Orthobunyavirus* genus specific real-time TaqMan RT-PCR when we noticed that cases were missed by the HyProbe in the real-time PCR although it could still be visualized on an agarose gel, suggesting the primers were sensitive but the probe area was different in some strains (Results not shown). Implementation of a genus specific PCR, designed to detect members of the Simbu serogroup (described in [24]) to screen cases from 2017 to2019 detected an additional 5/24 (20.8%) SHUV cases in 2018 and 2019 which were missed by the probes in the published assay. SHUV cases were identified in each year except in 2012 and 2017, while most cases were detected in 2010 (5/146, 3.4%), 2015 (5/199, 2.4%) followed by 2018 (4/140, 2.9%) accounting for 14/24, 58.3%, of the total cases (Table 1A).

### 3.1. SHUV Seasonality and Distribution.

**Figure 1:**
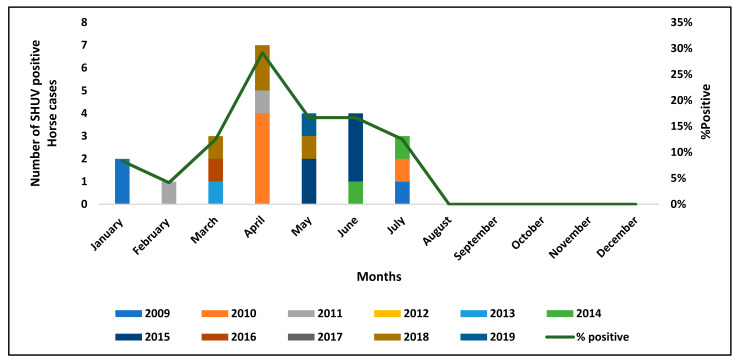
Graphical representation showing the seasonality of SHUV. The SHUV horse cases were detected from January to December between the years 2009 to 2019. The positivity rate is shown as percentages (%) and indicates the rate at which SHUV was detected in the different months throughout the years.

**Figure 2:**
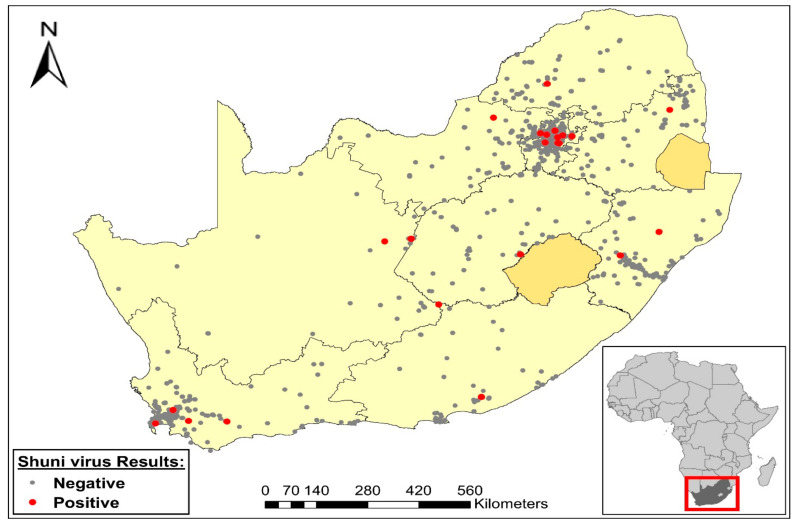
Map showing the distribution of SHUV cases in the different provinces across South Africa. The red dots represent the SHUV positive cases and the grey dots represent the negative cases.

Most SHUV cases occurred in the first half of the year between January and July with a peak in April (Figure 1). SHUV cases were identified in all nine provinces of SA (Figure 2) with most cases detected in Gauteng (9/24, 37.5%), followed by the Western Cape (4/24, 16.7%) (Table 1B).

### 3.2. Characteristics of SHUV Infected Horses

The sex of 18/24 of the SHUV positive cases was known of which 12/24 (50%) were females, 6/24 (25%) were male and the sex of 6/24 (25%) was not recorded (Table 2B). SHUV positive cases were detected in horses up to 20 years of age (Table 2C), with most cases occurring in young horses <5 y/o ((8/24, 33.3%). The greatest odds to test positive was in horses 11–15 years of age (OR 2.6 (0.70–7.9)).

### 3.3. Clinical Presentation of SHUV Horse Cases

The main syndromes investigated were neurological signs, death, and fever (Table 1C). Neurological signs were present in 21/24 (87.5%) of the cases and were significantly associated with SHUV infection (odds ratio (OR) 2.54, 95% confidence interval (CI) 0.75–8.56) (Table 2E). Death was reported in 11/24 (45.8%) of the cases and was also significantly associated with a positive SHUV test (OR 2.82, 95% CI 1.25–6.34) (Table 2D). Fever was detected in 7/24 (29.2%) of the SHUV cases, however, was not found to be significantly associated with SHUV relative to negative cases (OR 0.41, 95% CI 0.16–1.03) (Table 2D). Co-infections were detected with African horse sickness virus (AHS) and Middleburg virus (MIDV), in 2/5 (40%) of cases in 2010 and with West Nile virus (WNV) in 1/2 (50%) in 2014 (Table 1C). The neurological signs detected included ataxia (6/24, 25%), paralysis (3/24, 1.5%) and recumbence (4/24, 17%) (Table 2E). Non-neurological signs detected included anemia (1/24, 4.2%) and anorexia (2/24, 8.3%) however, these were not significantly associated with SHUV (OR 0.59, CI 0.08-4.42 and OR 0.44, CI 0.1-1.89 respectively) (Table 2F). Additional signs investigated but not detected in any of the SHUV positive cases, included blindness, seizures, paralysis, and abortions (Table 2E,F).

### 3.4. Phylogenetic Analysis

Maximum likelihood analysis based on the nucleocapsid (S) segment was used to compare the newly detected SHUV strains between 2018 and 2019 with the historic strains described in SA between 2009 and 2012 [25]. These were also compared to SHUV isolates from Nigeria and Israel and other members of the Simbu serogroup. All RT-PCR positive samples could be confirmed by sequencing to cluster with orthobunyaviruses in the Simbu serogroup based on the orthobunyavirus PCR fragment.

**Figure 3:**
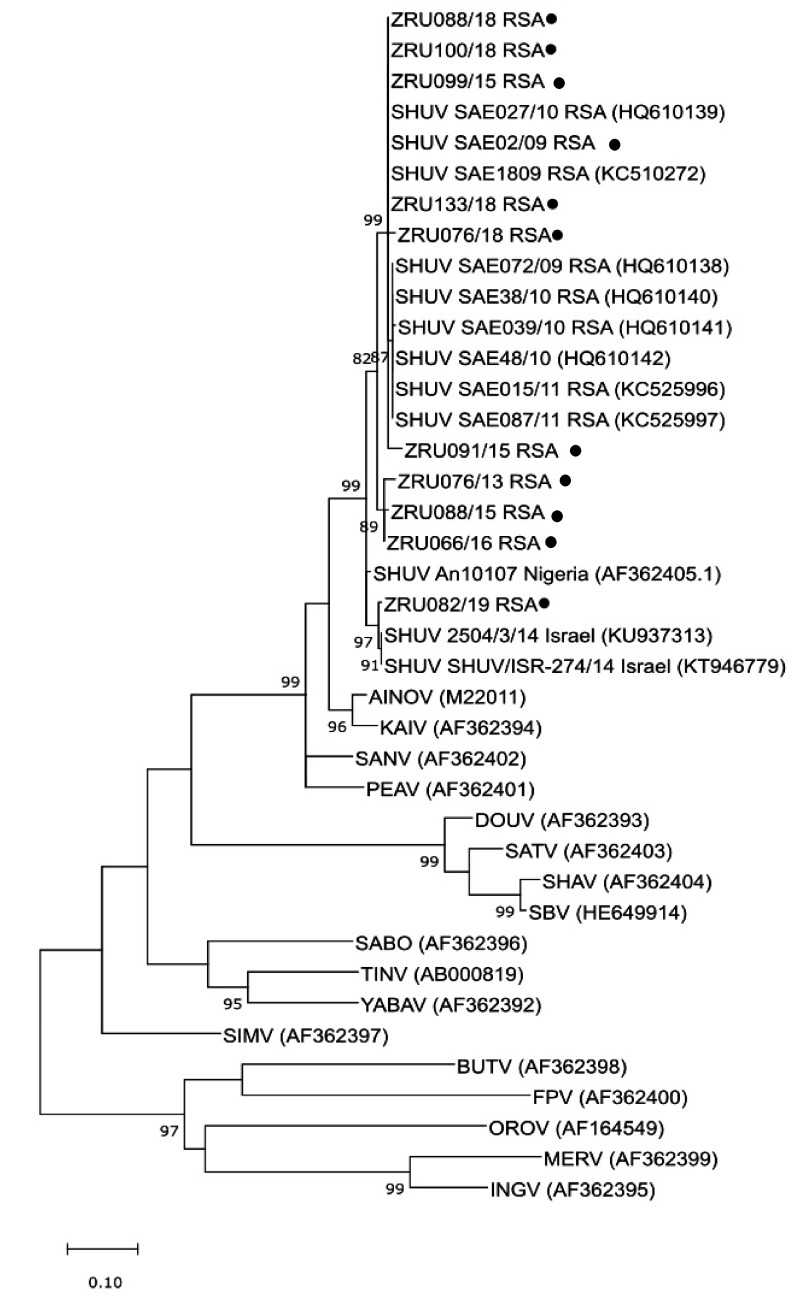
Phylogenetic analysis of SHUV positive horse cases based on a larger region of the nu-cleocapsid S-segment amplified with SHUV specific primers. The evolutionary rela-tionship was inferred by using the maximum likelihood method and Tamura-Nei model. The tree with the highest log likelihood (−3406.06) is shown. The bootstrap val-ues greater than 80% are shown next to the branches. The newly sequenced SHUV positive samples detected between 2013 and 2019 are indicated with black circles. South African strains from 2009–2012 (Van Eeden et al., 2013) are indicated with the prefix SHUV SAE. Simbu serogroup reference sequences were obtained from GenBank and the accession numbers are indicated in brackets next to the strain name: AINOV (Aino virus), AKAV (Akabane), BUTV (Buttonwillow virus), DOUV (Douglas virus), FPV (Faceys Paddock virus), INGV (Ingwavuma virus), KAIV (Kaikalurvirus), KAIRV (Kairi virus), MERV (Mermet virus), OROV (Oropouche virus), PEAV (Peaton virus), SABOV (Sabo virus), SANV (Sango virus), SATV (Sathuperi virus), SBV (Schmallen-berg virus), SHAV (Shamonda virus), SHUV (Shuni virus), SIMV (Simbu virus), TINV (Tinaroo virus), THIV (Thimiri), YABA (Yaba-7 virus). The SHUV GenBank accession numbers detected in the study: (SAE02/09 (MN937199), ZRU076/13 (MN901977), ZRU088/15 (MN901978), ZRU091/15 (MN901979), ZRU099/15 (MN901980), ZRU066/16 (MN901981), ZRU076/18 (MN901982), ZRU088/18 (MN901983), ZRU100/18 (MN901984), ZRU113/18 (MN901985), ZRU082/19 (MN937198).

Due to the short fragment length (152 bp) and conserved nature of the region targeted by the orthobunyavirus PCR, this was not ideal for resolving the evolutionary relationships and only used to confirm these viruses as orthobunyaviruses (results not shown). Therefore, phylogenetic analysis of a 327 bp fragment (position 317-643) was achieved by amplifying a larger region of the nucleocapsid (S) segment using the previously published SHUV specific primers by van Eeden et al. [25] in a conventional nested RT-PCR. The newly detected strains from 2013–2019 clustered with the 2009 SHUV horse isolate from SA (SAE18/09) and the other published horse strains from SA detected between 2009 and 2012 [17] (Figure 3). However, one of the samples (ZRU082/19), a horse from Gauteng, clustered with the SHUV strains from Israel. P-distance analysis of the larger region of the 2013–2019 strains were 98–99% similar to the SA strains, 96–97% similar to the Nigerian strain, and 94–95% similar to the Israeli strains.

### 3.5. Full Genome Analysis

**Figure 4:**
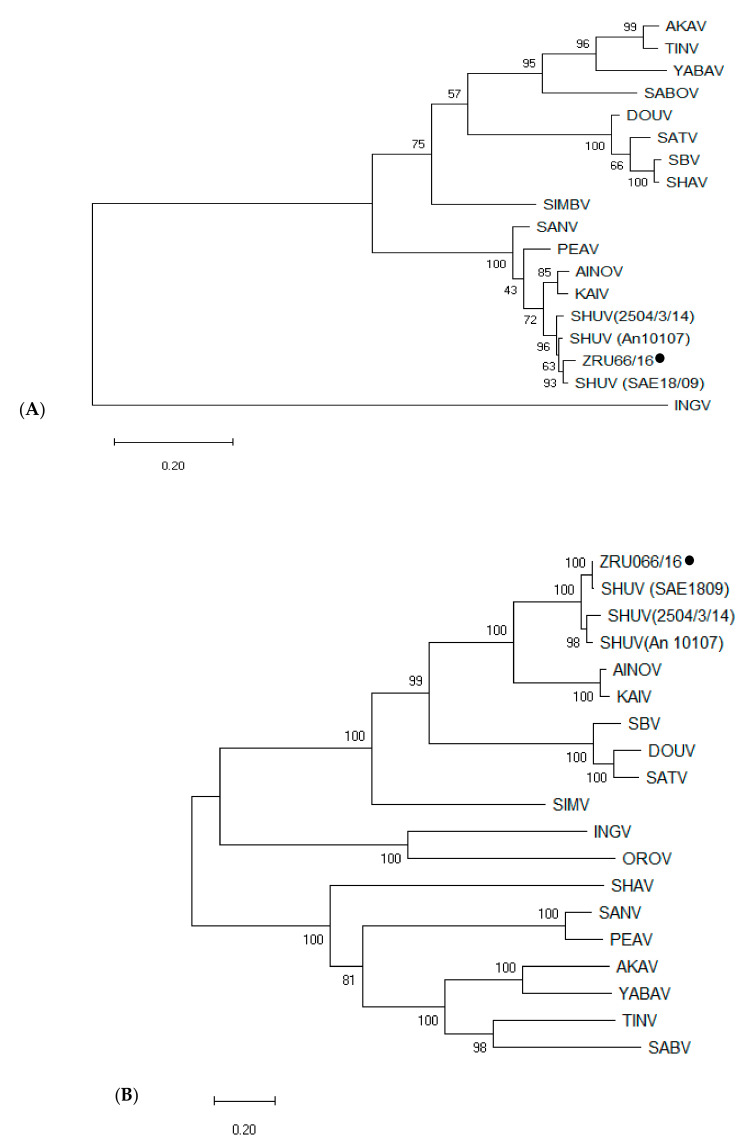

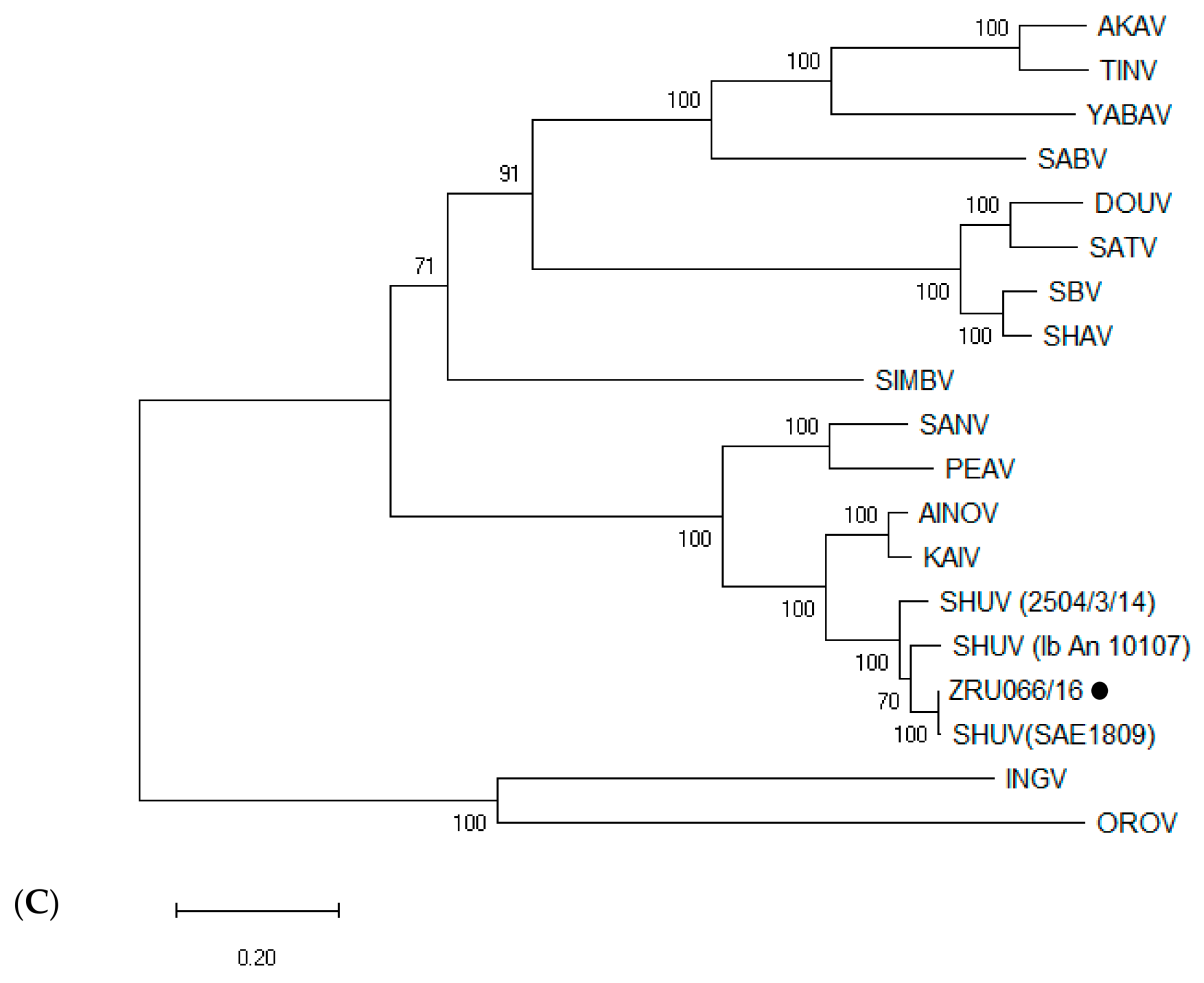
Phylogenetic relationship between the S (**A**), M (**B**) and L (**C**) segments of the SHUV (ZRU066/16) strain**.** Maximum-likelihood trees were constructed using the MEGA X Tamura-Nei substitution model (bootstrap 1000) with representative full genome sequences of other Simbu serogroup viruses. The newly sequenced SHUV strain (ZRU066/16) is indicated with a black circle. Simbu serogroup reference sequences were obtained from GenBank: AINOV (Aino virus,), AKAV (Akabane), DOUV (Douglas virus), INGV (Ingwavuma virus), KAIV (Kaikalur virus), OROV (Oropouche virus), PEAV (Peaton virus), SABOV (Sabo virus), SANV (Sango virus), SATV (Sathuperi virus), SBV (Schmallenberg virus), SHAV (Shamonda virus), SHUV (Shuni virus), SIMV (Simbu virus), TINV (Tinaroo virus), YABA (Yaba-7 virus). ZRU066/16 full genome GenBank accession numbers (MW729741, MW729742, MW729743).

The full genome sequence was determined from strain ZRU066/16, from the brain of a horse that suffered from severe neurological signs which was isolated on baby hamster kidney (BHK) cells. The virus caused a total destruction of the cells that could be observed as early as day two after inoculation. Once infected, the cells changed morphology and detached from the surface of the flask. The isolate was compared to available SHUV full genomes from South Africa, Nigeria and Israel and other members of the Simbu serogroup (Figure 4). The S segment of isolate ZRU066/16 was 863 nucleotides (nt) long with a 3′untranslated region (UTR) of 34 nt, 5′UTR of 127 nt and a NSs protein of 276 nt (Figure 4A). Phylogenetically, the S segment of ZRU066/16 clustered with the SHUV strains (SAE18/09, An10107), Aino (AINOV) and Kaikulur (KAIV) viruses with percentage identities of 98% (SHUV strains) and 94% (AINOV and KAIV), respectively. The M segment of isolate ZRU066/16 was 4347 nt with a 3′UTR of 23 nt and a 5′UTR of 132 nt (Figure 4B). The individual proteins (Gn, NSm and Gc) on the M segment were 254, 156, and 940 amino acids (aa) in length, respectively. Phylogenetically, the M segment of ZRU066/16 clustered with the SHUV strains (SAE18/09 (SA), An10107 (Nigeria) and 2504/3/14 (Israel) with 99, 95 and 94% similarity, respectively. The L segment of isolate ZRU066/16 was 6870 nt in length with a 3′UTR of 30 nt and a 5′UTR of 77 nt (Figure 4C). Phylogenetically, the L segment of ZRU066/16 clustered with the SHUV strains (SAE18/09 (SA), An10107 (Nigeria) and 2504/3/14 (Israel)) with percentage identities of 99, 93 and 93% respectively. The terminal sequences of the 3′and 5′UTR of all three ZRU066/16 genome segments were complementary to each other as is characteristic of the orthobunyaviruses.

## 4. Discussion

This study aimed to describe the epidemiology of SHUV by investigating the temporal and geographical distribution of SHUV in horses with febrile and/or neurological infections in SA over a period of 10 years. Horses have been shown to be sensitive sentinels for outbreaks of WNV neurological disease and may predict human outbreaks [32]. Since the reservoir host for SHUV is not known but cases of neurological disease have previously been described in horses, we hypothesize that horses could be used to detect SHUV activity and describe the epidemiology in endemic regions.

A real-time nested RT-PCR for SHUV has previously been described [25] and has been used to screen for SHUV cases since 2009, however in 2018 we noted that the SHUV specific real-time probes were unable to detect clinical strains although they could be detected by the primers when subjected to agarose gel electrophoresis. To avoid missing cases, this prompted us to change the screening method to a newly designed orthobunyavirus genus-specific TaqMan RT-PCR designed to detect all members of the Simbu serogroup (described in detail in Steyn et al. (in press)). Positive cases were amplified with both the orthobunyavirus TaqMan PCR (targeting 156 bp) and a SHUV conventional nested PCR (targeting 468 bp) based on the nucleocapsid (S) segment for sequence confirmation and molecular epidemiological analysis.

All cases were submitted with a case investigation form that included demographics and a clinical history including signs, vaccination status, location, age and breed. SHUV was most prevalent in horses in 2010 (5/146, 3.4%) and 2015 (5/199, 2.5%). These were both years of high rainfall with 2010 also coinciding with a Rift Valley Fever (RVF) outbreak in SA [33]. SHUV cases displayed clear arbovirus seasonality with all cases detected between the late summer to early winter season and towards the end of the rainy season in SA. These results correlate with arbovirus seasonality detected for other arboviruses such as AHSV and WNV [34] which may contribute to the reason that SHUV cases may have been missed in the past.

Cases were identified in all nine provinces. This may relate to areas where most horses reside, have easy accessibility to veterinary care and from where most specimens were received. This distribution may, however, be an under-representation of the true burden of SHUV in SA since cases are often sampled when severe neurological signs are displayed and may be late in the disease, therefore missing the viremia phase. The positive samples included whole blood samples (16/24 (66%)) mostly taken from live horses and post mortem tissue samples (8/24 (33%)) which included brain and spleen taken from dead horses. Case numbers may increase if IgM serological assays are developed. The specimens received depend on the number of specimens that are sent by veterinarians from across the country and do not necessarily indicate the true burden of SHUV in each province. Furthermore, SHUV infection was more prevalent in younger horses with the risk of infection decreasing with increasing age. This may be related to immunity being acquired in older horses in endemic regions.

All cases were submitted with case investigation forms indicating the demographics and clinical history as part of the surveillance program allowing us to determine if specific clinical signs can be associated with acute SHUV infections in horses. Neurological signs and death were strongly associated with SHUV infection, with 88% of cases having neurological signs, supporting our previous report that SHUV may be a missed cause of neurological infections in animals in SA [17]. This also correlated with recent reports in Israel that detected SHUV in neurological infections in cattle [20]. The fatality rate reported in this study was approximately 50%, indicating a positive association with SHUV infections. Although fewer SHUV cases than those caused by other neurotropic arboviruses were detected in SA, SHUV appears to have a higher fatality rate as opposed to 34% for WNV [32]. SHUV has also been reported to be associated with abortion and birth defects in livestock in Israel [21]. Therefore, SHUV should be considered as a differential diagnosis for abortion and fetal defects in Africa. Other signs detected in the SHUV cases included recumbency, ataxia, anemia, and paralysis.

Phylogenetic analysis based on the nucleocapsid (N) protein gene of the S-segment of the newly sequenced SHUV strains were compared to SHUV reference strains from SA, Nigeria, Israel and other members of the Simbu serogroup. The specimens were re-amplified using the SHUV specific conventional nested PCR without the probe which targeted a larger 463 bp region of the nucleocapsid gene. Maximum likelihood analysis clustered all the 2018 SHUV strains with each other as well as with the SHUV reference strains previously detected in SA [17], however the one sample from 2019 (ZRU082/19) clustered strongly with the Israeli reference strain. These results confirmed that the detected positive samples were all SHUV, but suggested that the 2019 strain was more similar to those that emerged in Israel than the strains previously detected in SA.

The SHUV samples were subjected to virus culture which resulted in one successful isolation. The isolate was from the brain of a horse (ZRU066/16) from KZN. The full genome analysis of ZRU066/16 showed that it was highly similar to the SA strain (SAE 18/09), however distinct differences were detected. The overall topology of the three segments displayed the common orthobunyavirus characteristics when compared to other genome studies [6,35]. Overall the SHUV strain ZRU066/16 grouped with the previously sequenced SA SHUV strain and was found to be closely related to AINO and KAIR viruses. Comparison of the ZRU066/16 strain to the other SHUV strains at the amino acid (aa) level showed some differences at each segment. The aa differences ranged between 2–3% for the S segment confirming the conserved nature of this segment. More variability was observed in the M segment with aa differences ranging between 0.6–6.2% with most differences observed in the prototype strain from Nigeria (An10107). The L segment which is the largest protein had the least overall conservation as the percentage differences were as high as 7.2%. However, fewer differences were observed in the SA strain (SAE18/09) with 0.6%, indicating a strong relation between the two stains.

The lack of epidemiological data and specific tests for SHUV in the past may have contributed to it being neglected over the past four decades since its first detection in Nigeria. Data presented here suggest that SHUV cases occurred annually, and despite being rare, were associated with severe neurological disease and death in horses in Africa and should be considered as a differential diagnosis for other arboviruses. More studies on SHUV as a cause of neurological and febrile disease in other species and potentially humans are underway to fully describe its epidemiology and contribution to human disease in Africa. A recent study conducted by our research group detected SHUV in CSF samples from hospitalized human patients [19]. These results suggest that SHUV has the potential to cause neurological diseases in humans. The cases are likely infected through mosquito bite rather than zoonotic transmission since most identified cases were children; nevertheless, further studies are warrant to determine epidemiological links between human and animal cases with detected animal cases potentially serving as an early warning for human disease outbreaks in the same region. Therefore, one health investigation of animals, humans, and vectors will assist in determining the risk of SHUV infection and its geographic range. The close relationship of recent South African strains to the Israeli strains does suggest that there is a risk for wider geographical spread with a significant risk for spread to other countries in the region and those where the vectors and reservoir host/s occur.

In conclusion, this study conducted over a ten-year period confirmed that SHUV in SA is associated with neurological and febrile illnesses with a high mortality rate in horses although it is relatively rare. More cases may be detected through the development of an IgM ELISA Furthermore, SHUV also poses a risk for transmission to other regions, likely through migratory birds, other reservoir hosts and/or infected vectors. SHUV should be investigated as a cause of neurological disease in animals and humans in other African countries and monitored for expansion to new regions such as Europe and the Middle East.

## Figures and Tables

**Table 1:** SHUV infection, co-infection, disease, and death in horses in South Africa between the years 2009 and 2019.

	No. (%) Horses											
Categories	2009	2010	2011	2012	2013	2014	2015	2016	2017	2018	2019	Total
**Total Horse Cases Sampled**												
	92	146	165	82	144	204	199	132	424	140	92	1820
A. **Number (n) of Confirmed SHUV Positive Cases (%) Ɨ**												
	3 (3.3)	5 (3.4)	2 (1.2)	0	1 (0.7)	2 (1)	5 (2.5)	1 (0.8)	0	4 (2.9)	1 (1.1)	24 (1.3)
**B. Provinces: n (%)**												
Gauteng	2 (2.2)	2 (1.4)	0	0	0	0	3 (1.5)	0	0	1 (0.7)	1 (1.1)	9 (0.5)
Limpopo	1 (1.1)	0	0	0	0	1 (0.5)	0	0	0	0	0	2 (0.1)
Mpumalanga	0	0	1 (0.6)	0	0	0	0	0	0	0	0	1 (0.05)
Free-State	0	0	0	0	0	1 (0.5)	0	0	0	0	0	1 (0.05)
Western Cape	0	0	1 (0.6)	0	1 (0.7)	0	0	0	0	2 (1.4)	0	4 (0.2)
Eastern Cape	0	0	0	0	0	0	1 (0.5)	0	0	0	0	1 (0.05)
Northern Cape	0	3 (2.1)	0	0	0	0	0	0	0	0	0	3 (0.2)
Kwa-Zulu Natal	0	0	0	0	0	0	1 (0.5)	1 (0.8)	0	0	0	2 (0.1)
North West	0	0	0	0	0	0	0	0	0	1 (0.7)	0	1 (0.05)
**C. Main Syndrome: n (%)**												
Death	3 (100)	1(20)	1 (50)	0	1 (100)	2 (100)	0	1 (100)		2 (50)		11(45.8)
Any neurological sign	3 (100)	4(80)	1 (50)	0	1 (100)	2 (100)	5 (100)	1 (100)		3 (75)	1 (100)	21 (87.5)
Fever	0	1 (20)	0	0	1 (100)	1 (50)	3 (60)	0	0		1(25)	7 (29.2)
Co-infection	0	2 (40)	0	0	0	1 (50)	0	0	0	0	0	3 (12.5)
Viruses		1 AHS				1 WNV						
		1 MIDV										

AHSV (African horse sickness virus), MIDV (Middleburg virus), SHUV (Shuni virus), WNV (West Nile virus). n (number of cases); % Percentage positive of total number of specimens tested. Percentage of total number of confirmed SHUV positive cases. Confirmed cases are those that tested positive by PCR.

**Table 2:** Demographics of the horses that were investigated with clinical samples that tested positive or negative for SHUV across South Africa between 2009 and 2019.

No. (%) Horses			
	SHUV Positive, *n* = 24	SHUV Negative, *n* = 1796	Crude OR (95% CI)
**A. Detection Method and Result**			
SHUV nested real-time PCR +	19/24 (79.2)	N/A	
Orthobunyavirus TaqMan PCR+	5/24 (20.8)	N/A	
**B. Sex**			
Male	6/24 (25)	739/1796 (41.1)	
Female	12/24 (50)	562/1796(31.3)	
Not specified	6/24 (25)	495/1796 (27.6)	
**C. Age in Years**			
<5	8/24 (33.3)	786/1796 (43.8)	1.00 (Reference)
6–10	6/24 (25)	331/1796 (18.4)	1.78 (0.61–5.18)
11–15	4/24 (16.7)	167/1796 (9.3)	2.6 (0.70–7.9)
16–20	1/24 (4.2)	124/1796 (6.9)	0.8 (0.1–6.4)
21–25	0/24 (0)	40/1796 (2.2)	0.0
26–30	0/24 (0)	18/1796 (1)	0.0
Not provided	5/24 (20.8)	330/1796 (18.4)	1.5 (0.48–4.59)
**D. Main Syndromes**			
Dead or euthanized	11/24 (45.8)	415/1796 (23.1)	2.82 (1.25–6.34)
Fever	7/24 (29.2)	806/1796 (44.9)	0.41 (0.16–1.03)
**E. Neurological Signs**			
Any neurological sign **	21/24 (87.5)	1318/1796 (73.4)	2.54 (0.75–8.56)
Ataxia	6/24 (25)	612/1796 (34.1)	0.64 (0.25–1.63)
Hind leg paralysis	0/24 (0)	72/1796 (4)	0
Paralysis	3/24 (12.5)	205/1796 (11.4)	0.11 (0.33–3.75)
Recumbency	4/24 (16.7)	251/1796 (14)	1.23 (0.42–3.63)
Seizure	0/24 (0)	89/1796 (5)	0
Tongue paralysis	0/24 (0)	17/1796 (0.9)	0
**F. Other Signs**			
Abortion	0/24 (0)	20/1796 (1.1)	0
Anemia	1/24 (4.2)	123/1796 (6.8)	0.59 (0.08–4.42)
Anorexia	2 /24 (8.3)	306/1796 (17)	0.44 (0.10–1.89)
Blindness	0/24 (0)	28/1796 (1.6)	0
Rectal prolapse	0/13 (0)	13/1796 (0.7)	0

+ Positive. Sex *p* = 0.13 (Fisher’s exact test). Age test for trend *p* = 0.764. ** Defined as the presence of any specific neurologic sign or the clinician indicated that it was a neurologic case without indicating specific neurologic signs.

## Data Availability

Additional data is available from the corresponding author.

## Supplementary Materials

The following are available online at https://www.mdpi.com/article/10.3390/v13050937/s1, Figure S1: title, Table S1: Summary of the SHUV positive horse cases (*n* = 24) detected between 2009-2019.
