# Supplementary material for: Epidemiology of Shuni Virus in Horses in South Africa
Source: Viruses. 2021 May 19;13(5):937. doi: 10.3390/v13050937 (PMC8158722; doi:10.3390/v13050937)
Supplement: Supplementary file 1 [file viruses-13-00937-s001.zip › viruses-1116235-supplementary.pdf]

Supplementary Table S1. Summary of the SHUV positive horse cases (*n* = 24) detected between 2009-2019

Sample ID	Year	Province	Sample Type	Main Syndrome	Detection Method	Outcome	GenBank Accession Number	Published
**SAE02/09**	2009	Limpopo	Brain	Neurological	SHUV specific HybProbe nested real-time RT-PCR	Fatal	MN937199	Current study
**SAE18/09**	2009	Gauteng	Brain	Neurological	SHUV specific HybProbe nested real-time RT-PCR	Fatal	KC510272	(van Eeden et al., 2012)
**SAE72/09**	2009	Gauteng	Brain	Neurological	SHUV specific HybProbe nested real-time RT-PCR	Fatal	HQ610138	(van Eeden et al., 2012)
**SAE27/10**	2010	Gauteng	Whole blood	Neurological	SHUV specific HybProbe nested real-time RT-PCR	Fatal	HQ60139	(van Eeden et al., 2012)
**SAE38/10**	2010	Northern Cape	Whole blood	Fever	SHUV specific HybProbe nested real-time RT-PCR	Survived	HQ60140	(van Eeden et al., 2012)
**SAE39/10**	2010	Northern Cape	Whole blood	Neurological	SHUV specific HybProbe nested real-time RT-PCR	Survived	HQ60141	(van Eeden et al., 2012)
**SAE48/10**	2010	Northern Cape	Whole blood	Neurological	SHUV specific HybProbe nested real-time RT-PCR	Survived	HQ60142	(van Eeden et al., 2012)

**SAE109/10***	2010	Gauteng	Whole blood	Neurological	SHUV specific HybProbe nested real-time RT-PCR	Survived	-	-
**SAE15/11**	2010	Mpumalanga	Whole blood	Acute Death	SHUV specific HybProbe nested real-time RT-PCR	Fatal	KC525996	(van Eeden et al., 2012)
**SAE87/11**	2011	Western Cape	Whole blood	Neurological	SHUV specific HybProbe nested real-time RT-PCR	Survived	KC525997	(van Eeden et al., 2012)
**ZRU076/13**	2013	Western Cape	Brain	Fever and Neurological	SHUV specific HybProbe nested real-time RT-PCR	Fatal	MN901977	Current study
**ZRU167/14***	2014	Free-State	Spleen	Fever and Neurological	SHUV specific HybProbe nested real-time RT-PCR	Fatal	-	-
**ZRU189/14***	2014	Limpopo	Spleen	Neurological	SHUV specific HybProbe nested real-time RT-PCR	Fatal	-	-
**ZRU088/15**	2015	Kwa-Zulu Natal	Whole blood	Fever and Neurological	SHUV specific HybProbe nested real-time RT-PCR	Survived	MN901978	Current study
**ZRU091/15**	2015	Gauteng	Whole blood	Neurological	SHUV specific HybProbe nested real-time RT-PCR	Survived	MN901979	Current study
**ZRU099/15**	2015	Eastern Cape	Whole blood	Neurological	SHUV specific HybProbe nested real-time RT-PCR	Survived	MN901980	Current study
**ZRU103/15***	2015	Gauteng	Whole blood	Fever and Neurological	SHUV specific HybProbe nested real-time RT-PCR	Survived	-	-

**ZRU111/15***	2015	Gauteng	Whole blood	Fever and Neurological	SHUV specific HybProbe nested real-time RT-PCR	Survived	-	-
**ZRU066/16**	2016	Kwa-Zulu Natal	Brain	Neurological	SHUV specific HybProbe nested real-time RT-PCR	Fatal	MN901981	Current study
**ZRU076/18**	2018	North West	Serum	Neurological	Orthobunyavirus genus specific real-time TaqMan RT-PCR	Fatal	MN901982	Current study
**ZRU088/18**	2018	Western Cape	Whole blood	Neurological	Orthobunyavirus genus specific real-time TaqMan RT-PCR	Survived	MN901983	Current study
**ZRU100/18**	2018	Gauteng	Whole blood	Neurological	Orthobunyavirus genus specific real-time TaqMan RT-PCR	Fatal	MN901984	Current study
**ZRU113/18**	2018	Western Cape	Brain	Neurological	Orthobunyavirus genus specific real-time TaqMan RT-PCR	Fatal	MN901985	Current study
**ZRU082/19**	2019	Gauteng	Whole blood	Fever	Orthobunyavirus genus specific real-time TaqMan RT-PCR	Survived	MN937198	Current study

(*) Sequencing unsuccessful, EDTA (Ethylenediaminetetraacetic acid), RT (Reverse transcription), PCR (Polymerase chain reaction), SHUV (Shuni virus)
